# PREVALENCE OF AND ATTITUDES TOWARD MARIJUANA: THE CASE FOR CHALDEAN AMERICANS IN MICHIGAN

**DOI:** 10.51894/001c.122840

**Published:** 2024-08-30

**Authors:** Anthony Mansour, Anthony Cholagh, Bianca Elias, Angelina Selou, Carrie Nazaroff, Florence Dallo

**Affiliations:** 1 College of Osteopathic Medicine Michigan State University

## 20

In the last decade, several US states, including Michigan, have legalized recreational marijuana. Michigan, home to around 150,000 Chaldeans—originating from northern Iraq and often associated with the Eastern Rite Catholic Church—has seen little research on the impact of marijuana legalization on this community. This study aimed to explore Chaldean attitudes towards medicinal and recreational marijuana, estimate usage prevalence, examine the link between attitudes and religiosity, and evaluate Chaldean involvement in marijuana distribution.

The survey, conducted through Qualtrics, comprised internally generated and standardized questions, taking about 10 minutes to complete. Eligible participants were self-identified Chaldeans over 18 years old. The survey, distributed through Chaldean Facebook groups, the Chaldean Community Foundation, Student Associations, and Churches, ensured anonymity and voluntary participation.

From March 20th to April 20th, 2022, 901 respondents participated, with 52.9% aged 21-29 and 62.9% female. Notably, 44% supported recreational marijuana legalization, with 26% of supporters attending church weekly. Regarding medicinal marijuana, 75% supported its legalization, and 44.4% of supporters attended church weekly. Nearly half of the respondents (49.3%) reported past marijuana use, primarily for recreation (75.3%). In terms of industry involvement, 11.3% admitted participation, and 35.6% of those disclosed selling marijuana, with 14 selling at state-licensed dispensaries and 8 at state-licensed wholesale.

The results underscore the diversity in Chaldean attitudes and involvement with marijuana, potentially contributing to tensions between the Church and the community. However, the findings may not be universally applicable to Chaldean Americans in other states. Future studies should explore evolving attitudes and behaviors, incorporating qualitative research for deeper insights.

